# Supplementary material for: Hybrid Models and Biological Model Reduction with PyDSTool
Source: PLoS Comput Biol. 2012 Aug 9;8(8):e1002628. doi: 10.1371/journal.pcbi.1002628 (PMC3415397; doi:10.1371/journal.pcbi.1002628)
Supplement: Text S4 — Complete source code for the PyDSTool package (version 0.88.120504). Includes API documentation and help files linking to web pages. This file is identical to the current public release on Sourceforge.net. (ZIP) [file pcbi.1002628.s004.zip › PyDSTool/html/PyDSTool.Generator.Radau_ODEsystem'.Radau_ODEsystem-class.html]

xml version="1.0" encoding="ascii"?


PyDSTool.Generator.Radau\_ODEsystem'.Radau\_ODEsystem


| Home | Trees | Indices | Help | PyDSTool |
| --- | --- | --- | --- | --- |

Package PyDSTool ::
Package Generator ::
Module Radau\_ODEsystem' ::
Class Radau\_ODEsystem

[hide private]

[frames] | no frames]

# Class Radau\_ODEsystem

source code

```
           object --+            
                    |            
baseclasses.Generator --+        
                        |        
       baseclasses.ctsGen --+    
                            |    
         ODEsystem'.ODEsystem --+
                                |
                               Radau_ODEsystem
```

---

Wrapper for Radau integrator (with support for differential-algebraic
equations).

Uses C target language only for functional specifications


|  |  |
| --- | --- |
| Instance Methods | [hide private] |

|  |  |
| --- | --- |
| AuxVars(self, t, xdict, pdict=None, asarray=True)  asarray is an unused, dummy argument for compatibility with Model.AuxVars | source code |

|  |  |
| --- | --- |
| Jacobian(self, t, xdict, pdict=None, asarray=True)  asarray is an unused, dummy argument for compatibility with Model.Jacobian | source code |

|  |  |
| --- | --- |
| JacobianP(self, t, xdict, pdict=None, asarray=True)  asarray is an unused, dummy argument for compatibility with Model.JacobianP | source code |

|  |  |
| --- | --- |
| MassMatrix(self, t, xdict, pdict=None, asarray=True)  asarray is an unused, dummy argument for compatibility with Model.MassMatrix | source code |

|  |  |
| --- | --- |
| Rhs(self, t, xdict, pdict=None, asarray=True)  asarray is an unused, dummy argument for compatibility with Model.Rhs | source code |

|  |  |
| --- | --- |
| \_\_del\_\_(self) | source code |

|  |  |
| --- | --- |
| \_\_init\_\_(self, kw)  Use the nobuild key to postpone building of the library, e.g. | source code |

|  |  |
| --- | --- |
| \_ensure\_inputs(self, force=False) | source code |

|  |  |
| --- | --- |
| \_ensure\_solver(self, pars=None) | source code |

|  |  |
| --- | --- |
| \_prepareEventSpecs(self) | source code |

|  |  |
| --- | --- |
| compileLib(self, libsources=`[``]`, libdirs=`[``]`)  compileLib generates a python extension DLL with integrator and vector field compiled and linked. | source code |

|  |  |
| --- | --- |
| compute(self, trajname, dirn=`'``f``'`, ics=None)  This is an abstract class. | source code |

|  |  |
| --- | --- |
| forceLibRefresh(self)  forceLibRefresh should be called after event contents are changed, or alterations are made to the right-hand side of the ODEs. | source code |

|  |  |
| --- | --- |
| makeLib(self, libsources=`[``]`, libdirs=`[``]`, include=`[``]`)  makeLib calls makeLibSource and then the compileLib method. | source code |

|  |  |
| --- | --- |
| makeLibSource(self, include=`[``]`)  makeLibSource generates the C source for the vector field specification. | source code |

**Inherited from `ODEsystem'.ODEsystem`**:
`__getstate__`,
`__setstate__`,
`addMethods`,
`checkInitialConditions`,
`cleanupMemory`,
`haveJacobian`,
`haveJacobian_pars`,
`haveMass`,
`prepDirection`,
`set`,
`validateICs`

**Inherited from `baseclasses.ctsGen`**:
`validateSpec`

**Inherited from `baseclasses.Generator`**:
`__copy__`,
`__deepcopy__`,
`__repr__`,
`__str__`,
`addEvtPars`,
`checkArgs`,
`contains`,
`get`,
`getEventTimes`,
`getEvents`,
`info`,
`query`,
`resetEventTimes`,
`resetEvents`,
`setEventICs`,
`showAuxFnSpec`,
`showAuxSpec`,
`showEventSpec`,
`showSpec`

**Inherited from `baseclasses.Generator`** (private):
`_addEvents`,
`_auxfn_getindex`,
`_auxfn_globalindepvar`,
`_auxfn_heav`,
`_auxfn_if`,
`_auxfn_initcond`,
`_generate_ixmaps`,
`_infostr`,
`_kw_process_algparams`,
`_kw_process_allvars`,
`_kw_process_dispatch`,
`_kw_process_events`,
`_kw_process_fnspecs`,
`_kw_process_ics`,
`_kw_process_ignorespecial`,
`_kw_process_inputs`,
`_kw_process_pars`,
`_kw_process_pdomain`,
`_kw_process_reuseterms`,
`_kw_process_system`,
`_kw_process_target`,
`_kw_process_tdata`,
`_kw_process_tdomain`,
`_kw_process_tstep`,
`_kw_process_ttype`,
`_kw_process_varspecs`,
`_kw_process_vfcodeinserts`,
`_kw_process_xdomain`,
`_kw_process_xtype`,
`_makeBoundsEvents`,
`_register`,
`_set_for_hybrid_DS`

**Inherited from `object`**:
`__delattr__`,
`__getattribute__`,
`__hash__`,
`__new__`,
`__reduce__`,
`__reduce_ex__`,
`__setattr__`


|  |  |
| --- | --- |
| Class Variables | [hide private] |

\_paraminfo = `{'DAEstructureM1': '', 'DAEstructureM2': '', 'ato...`

**Inherited from `ODEsystem'.ODEsystem`** (private):
`_needKeys`,
`_optionalKeys`,
`_validKeys`

**Inherited from `baseclasses.Generator`** (private):
`_querykeys`


|  |  |
| --- | --- |
| Properties | [hide private] |

**Inherited from `object`**:
`__class__`


|  |  |
| --- | --- |
| Method Details | [hide private] |

|  |  |
| --- | --- |
| AuxVars(self, t, xdict, pdict=None, asarray=True) | source code |

asarray is an unused, dummy argument for compatibility with
Model.AuxVars

Overrides: ODEsystem'.ODEsystem.AuxVars

|  |  |
| --- | --- |
| Jacobian(self, t, xdict, pdict=None, asarray=True) | source code |

asarray is an unused, dummy argument for compatibility with
Model.Jacobian

Overrides: ODEsystem'.ODEsystem.Jacobian

|  |  |
| --- | --- |
| JacobianP(self, t, xdict, pdict=None, asarray=True) | source code |

asarray is an unused, dummy argument for compatibility with
Model.JacobianP

Overrides: ODEsystem'.ODEsystem.JacobianP

|  |  |
| --- | --- |
| Rhs(self, t, xdict, pdict=None, asarray=True) | source code |

asarray is an unused, dummy argument for compatibility with
Model.Rhs

Overrides: ODEsystem'.ODEsystem.Rhs

|  |  |
| --- | --- |
| \_\_del\_\_(self)  *(Destructor)* | source code |

Overrides: baseclasses.Generator.\_\_del\_\_

|  |  |
| --- | --- |
| \_\_init\_\_(self, kw)  *(Constructor)* | source code |

Use the nobuild key to postpone building of the library, e.g. in order
to provide additional build options to makeLibSource and compileLib
methods or to make changes to the C code by hand. No build options can be
specified otherwise.

Overrides: object.\_\_init\_\_

|  |  |
| --- | --- |
| compileLib(self, libsources=`[``]`, libdirs=`[``]`) | source code |

```
compileLib generates a python extension DLL with integrator and vector
field compiled and linked.

libsources list allows additional library sources to be linked.
libdirs list allows additional directories to be searched for
  precompiled libraries.
```

|  |  |
| --- | --- |
| compute(self, trajname, dirn=`'``f``'`, ics=None) | source code |

This is an abstract class.

Overrides: ODEsystem'.ODEsystem.compute: *(inherited documentation)*

|  |  |
| --- | --- |
| forceLibRefresh(self) | source code |

forceLibRefresh should be called after event contents are changed, or
alterations are made to the right-hand side of the ODEs.

Currently this function does NOT work!

|  |  |
| --- | --- |
| makeLib(self, libsources=`[``]`, libdirs=`[``]`, include=`[``]`) | source code |

makeLib calls makeLibSource and then the compileLib method. To
postpone compilation of the source to a DLL, call makelibsource()
separately.

|  |  |
| --- | --- |
| makeLibSource(self, include=`[``]`) | source code |

makeLibSource generates the C source for the vector field
specification. It should be called only once per vector field.

  


|  |  |
| --- | --- |
| Class Variable Details | [hide private] |

### \_paraminfo

Value:
:   ```
    {'DAEstructureM1': '',
     'DAEstructureM2': '',
     'atol': 'Absolute error tolerance.',
     'check_aux': 'Switch',
     'extraspace': '',
     'fac1': 'Parameter for step size selection; the new step size is chos
    en subject to the restriction  fac1 <= new_step/old_step <= fac2. Defa
    ult value is 1.0.',
    ...
    ```

  


| Home | Trees | Indices | Help | PyDSTool |
| --- | --- | --- | --- | --- |

|  |  |
| --- | --- |
| Generated by Epydoc 3.0.1 on Fri May 4 15:24:06 2012 | http://epydoc.sourceforge.net |
